# Supplementary material for: Giant intrinsic circular dichroism of prolinol-derived squaraine thin films
Source: Nat Commun. 2018 Jun 20;9:2413. doi: 10.1038/s41467-018-04811-7 (PMC6010436; doi:10.1038/s41467-018-04811-7)
Supplement: Supplementary file 1 — Supplementary Information [file 41467_2018_4811_MOESM1_ESM.pdf]

Supplementary Information:
Giant Intrinsic Circular Dichroism of Prolinol-Derived Squaraine Thin Films

Schulz *et al.*

Supplementary Note 1: Synthesis and Analytical Data of (*S,S*)- and (*R,R*)-ProSQ-C16

We have obtained the two enantiomers of a prolinol-functionalized squaraine compound (*S,S*)-ProSQ-C16 and (*R,R*)-ProSQ-C16 via an ex-chiral pool synthesis. Full details of the synthetic procedure have been published for the (*S,S*)-enantiomer previously by Schulz *et al.*.[1] The synthetic approach is outlined briefly in the following:

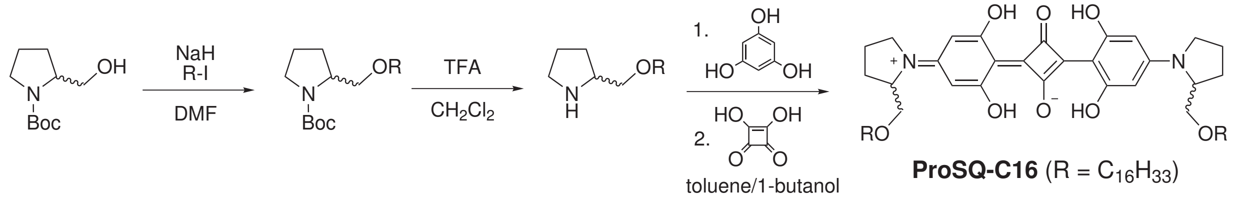

Supplementary Figure 1: Reaction scheme to obtain ProSQ-C16.

Boc-protected L- and D-prolinol are commercially available as chiral pool reactants in 97 % and 99 % enantiopurity, respectively, and used as received. Through a Williamson ether synthesis with an alkyl halide (here: alkyl = R = hexadecyl = C16) and subsequent deprotection the enantiopure amine precursors are obtained. These are further reacted in a one-pot two-step catalyst-free condensation with phloroglucinol firstly, and half an equivalent of squaric acid secondly, under azeotropic removal of evolving water. The crude products precipitate from the cooled reaction mixture and are collected by filtration in yields close to 50 %. In addition to the previously described procedure, the compounds were after recrystallization purified by column chromatography on silica gel (Merck, pore size 40 to 63 µm) with dichloromethane as mobile phase. Both compounds were analytically pure in nuclear magnetic resonance (NMR) and mass spectroscopic characterization. However, the optical purity ultimately depends on the enantiomeric purity of the chiral pool reactant. Unfortunately, the (*S,S*)-ProSQ-C16 has an admixture of 2 % of both the (*R,R*)-enantiomer and the (*R,S*)-diastereomer, which was evidenced by analytical chiral high-performance liquid chromatography (HPLC, DAICEL Chiralpak IA, *n*-hexane:ethanol 75:25, 1 mL/min). Full

analytical details are given in the following:

***(S,S)*-ProSQ-C16**

2,4-Bis[4-((*S*))-2-(hexadecyloxymethyl)-pyrrolidone-2,6-dihydroxyphenyl]squaraine

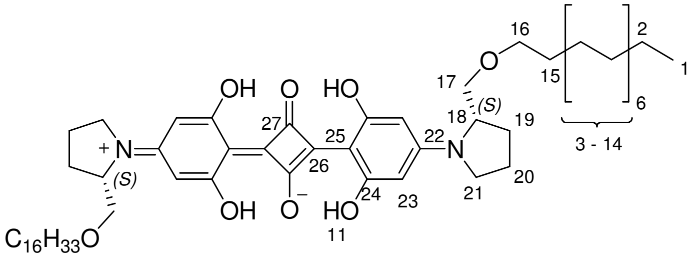

Yield: 25 %

Molecular formula: $C_{58}H_{92}N_2O_8H$

Molar mass: 945.38 g/mol

^{1}H NMR (Bruker Advance): (500 MHz, $CDCl_3$, RT) δ [ppm] = 11.01 (s, 4 H, H-11), 5.78 (s, 4 H, H-23), 4.10 – 4.02 (m, 2 H, H-18), 3.57 – 3.29 (m, 12 H, H-16, H-17, H-21), 2.17 – 1.96 (m, 8 H, H-19, H-20), 1.59 – 1.50 (m, 4 H, H-15), 1.35 – 1.19 (m, 52 H, H-2 – H-14), 0.88 (t, J = 6.9 Hz, 6 H, H-1).

^{13}C NMR (Bruker Advance): (126 MHz, $CDCl_3$, RT) δ [ppm] = 181.4 (C-27), 162.9 (C-24), 161.8 (C-22), 156.9 (C-26), 103.0 (C-25), 94.8 (C-23), 71.9 (C-16), 70.4 (C-17), 59.0 (C-18), 49.1 (C-21), 32.1/29.9/29.8/29.8/29.8/29.8/29.8/29.6/29.5/28.6/26.3/22.9/22.9 (C-2 – C-15, C-19, C-20), 14.3 (C-1).

ESI$^+$ HRMS (Bruker microTOF-Q): m/z calcd for $C_{58}H_{92}N_2O_8H$ $[M+H]^+$: 945.6926, found: 945.6932.

Elemental analysis (Heraeus Vario EL): $C_{58}H_{92}N_2O_8$ calcd C: 73.69, H: 9.81, N: 2.96; found: C: 73.40, H: 9.87, N: 2.96.

UV-Vis (chloroform): $\lambda_{max} = 646$ nm, $\epsilon = 344{,}200\,M^{-1}cm^{-1}$

R_f factor (dichloromethane): 0.9

(*R,R*)-ProSQ-C16

2,4-Bis[4-((*R*))-2-(hexadecyloxymethyl)-pyrrolidone-2,6-dihydroxyphenyl]squaraine

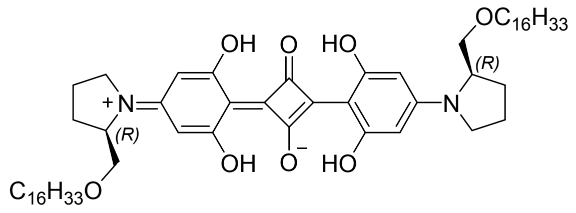

Yield: 25 %

Molar mass: $C_{58}H_{92}N_2O_8H$ 945.38 g/mol

All other analytical data of the (*R,R*)-enantiomer coincide with those of the (*S,S*)-enantiomer.

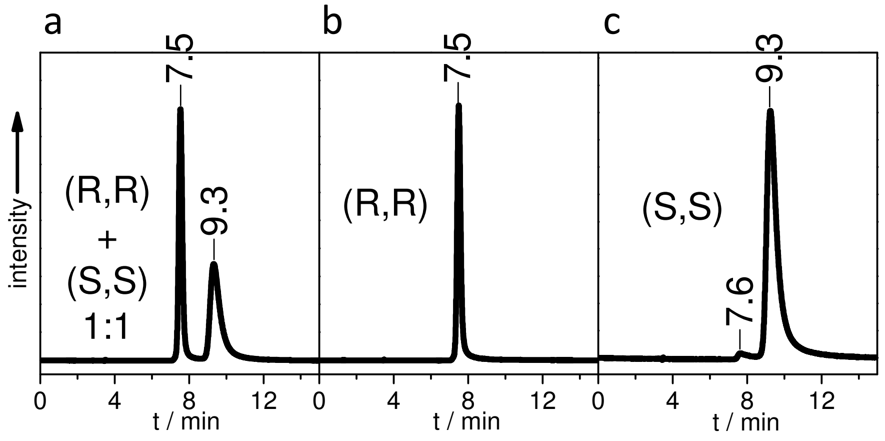

Supplementary Figure 2: Chromatograms of analytical chiral HPLC separations of the synthesized proline-derived chiral squaraines ProSQ-C16. (a) 1:1 mixture of (*R,R*)- and (*S,S*)-ProSQ-C16; (b) pure (*R,R*)-ProSQ-C16 (integral area 100 %); (c) (*S,S*)-ProSQ-C16 with impurity (integral area ratio approx. 98 %:2 %). Parameters: stationary phase: DAICEL Chiralpak IA, 5 µm, 4.6 mm × 250 mm; mobile phase: n-hexane/ethanol 75:25; injection volume: 1 µL; temperature: 25 °C; flow rate: 1 mL/min; detection: UV/Vis, $\lambda = 640$ nm.

Supplementary Note 2: Optical Purity Determination by Chiral HPLC

We attribute the lower CD value of the (*S,S*)-ProSQ-C16 to the presence of small quantities of the other enantiomer or diastereomers. We deduce this difference of otherwise both analytically pure ProSQ-C16 enantiomers from the different optical purities of the starting materials used for the syntheses. (*R,R*)-ProSQ-C16 which is derived from 99 % pure 1-Boc-D-prolinol (Fluorochem, ORD: +50.9° for 1.3 % in $CHCl_3$) performs superior with regard to optical activity. (*S,S*)-ProSQ-C16 on the other hand is synthesized from 97 % pure 1-Boc-L-prolinol (Fluorochem, ORD: −49.1° in $CHCl_3$) and performs worse.

In order to reinforce this assumption we utilize analytical chiral HPLC (Knaur system, Azura series with photodiode array detector DAD 6.1L). The results of the successful analytical chiral HPLC separations of a mixture of (*S,S*- and (*R,R*)-ProSQ-C16 and for both synthesized enantiomers separately using a DAICEL Chiralpak IA chiral stationary phase are shown as chromatograms in Fig. 2. For the mixture (a) both compounds are well separated and only two prominent UV-active peaks with very little tailing, which exhibit the same UV-Vis spectra, are observed. (*R,R*)-ProSQ-C16 (b) shows only a single sharp peak with 100 % integral ratio confirming its high optical purity. In the chromatogram of (*S,S*)-ProSQ-C16 (c) an impurity peak with a little shoulder and an integral area of at least 2 % is observed at the same retention time as (*R,R*)-ProSQ-C16 with broader tailing. We assign this broadened impurity with the same UV-Vis absorption to both the (*R,R*)-enantiomer as well as the (*R,S*)-diastereomer (*meso* compound).

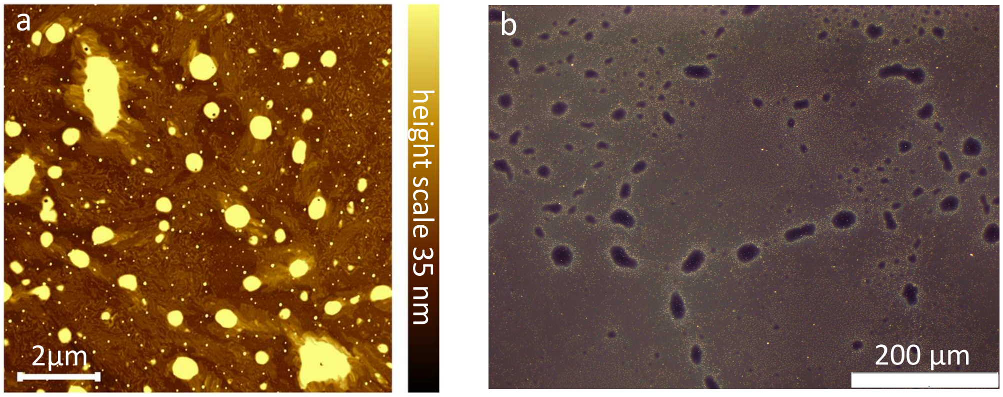

Supplementary Figure 3: (a) AFM image in intermittent contact mode (JPK NanoWizard) and (b) optical dark-field microscopy image (Olympus BX41) of a ProSQ-C16 thin film annealed at 240 °C. Substantial dewetting is noticeable disrupting the absorbance as well as circular dichroic properties of the sample.

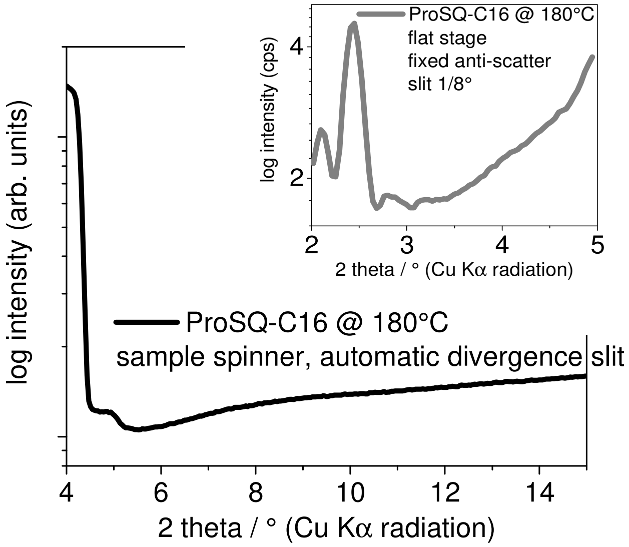

Supplementary Figure 4: X-ray diffraction pattern measured in Bragg-Brentano geometry (theta-2-theta scan) with a sample spinner to exclude effects from possible in-plane orientation (PANalytical X'PertPro MPD diffractometer with PIXcel detector, Cu Kα radiation, automatic divergence slit, fixed 10 mm incident beam mask, 6000 seconds per step, step size 0.039°) of ProSQ-C16 thin film annealed at 180 °C. The steep rise in the pattern for 2 θ angles smaller than 5° is caused by overshooting of the sample. To address this issue, a flat stage sample holder was used instead and an additional fixed 1/8° anti-scatter slit was mounted at the incident optics, and the theta-2-theta scan was repeated for small incident angles, see inset. A faint (4 counts per second) and broad peak becomes visible at 2 θ = 2.4° which corresponds to a d-spacing of 3.6 nm. The signal most likely originates from the regular height levels detected by AFM to be 3 nm, see Fig. 2.

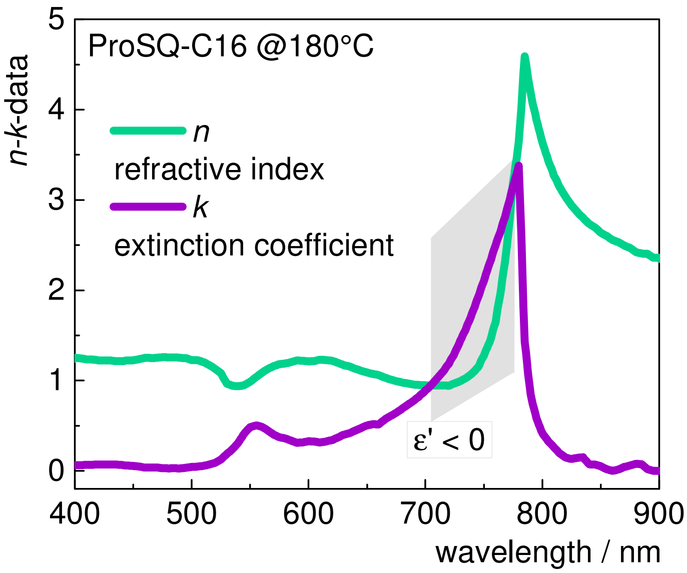

Supplementary Figure 5: Effective (isotropic, valid for both enantiomers) optical constants of ProSQ-C16 thin films annealed at 180 °C, sample thickness around 23 nm. The shaded area marks the spectral region where the real part of the dielectric function $\epsilon' = n^2 - k^2$ is negative.

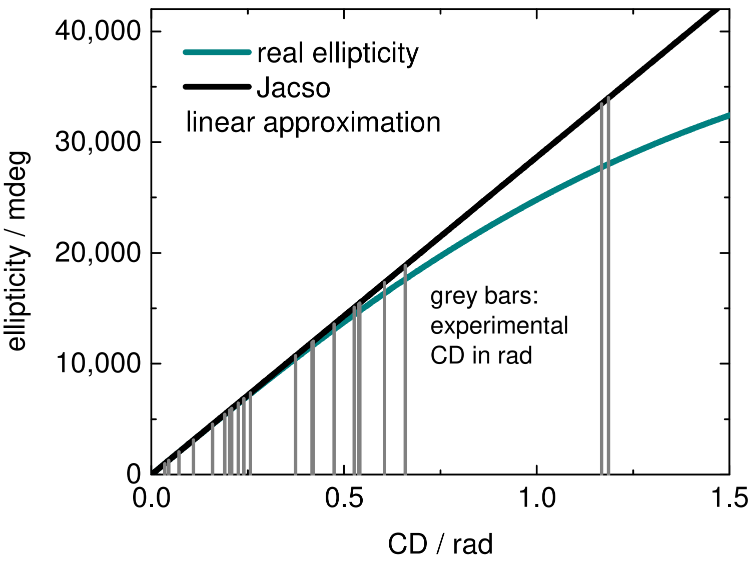

Supplementary Figure 6: Ellipticity calculated from CD in radians according to Eq. (3) (cyan line) and linear "Jasco" approximation for ellipticity according to Eq. (4) (black line). The grey bars denote actually measured CD values for ProSQ-C16 samples annealed at 180 °C and 210 °C.

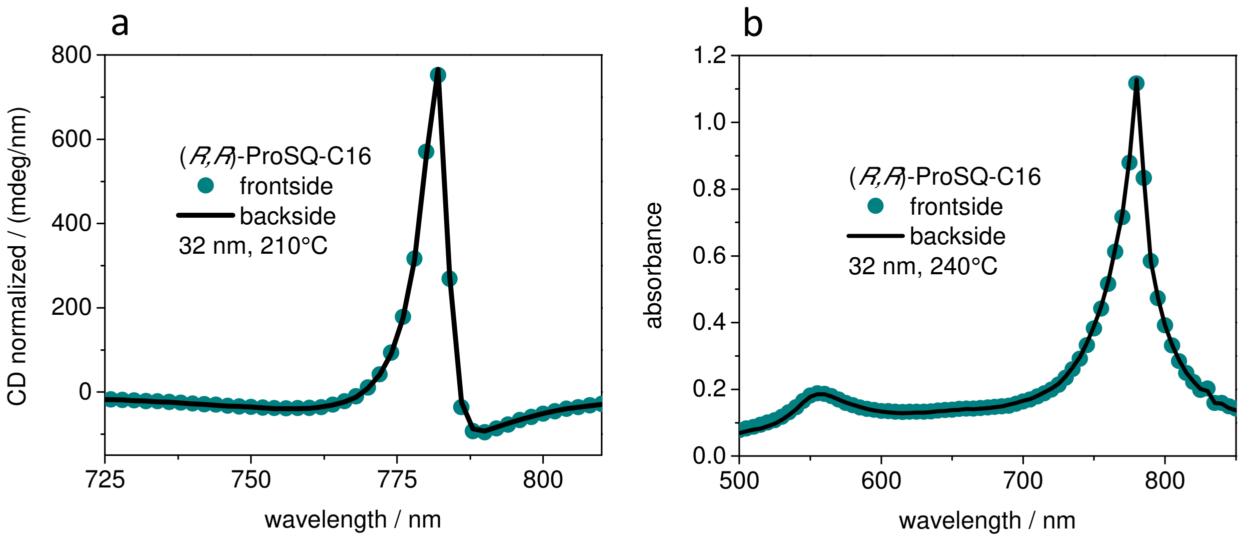

Supplementary Figure 7: (a) Thickness normalized CD extracted from Mueller matrix scans recorded from both sides of a 32 nm thick (*R*,*R*)-ProSQ-C16 thin film sample annealed at 210 °C and (b) corresponding absorbance spectra of the sample. "Backside" (black line) refers to measuring through the glass while "frontside" (cyan circles) is the usual way for measuring all samples.

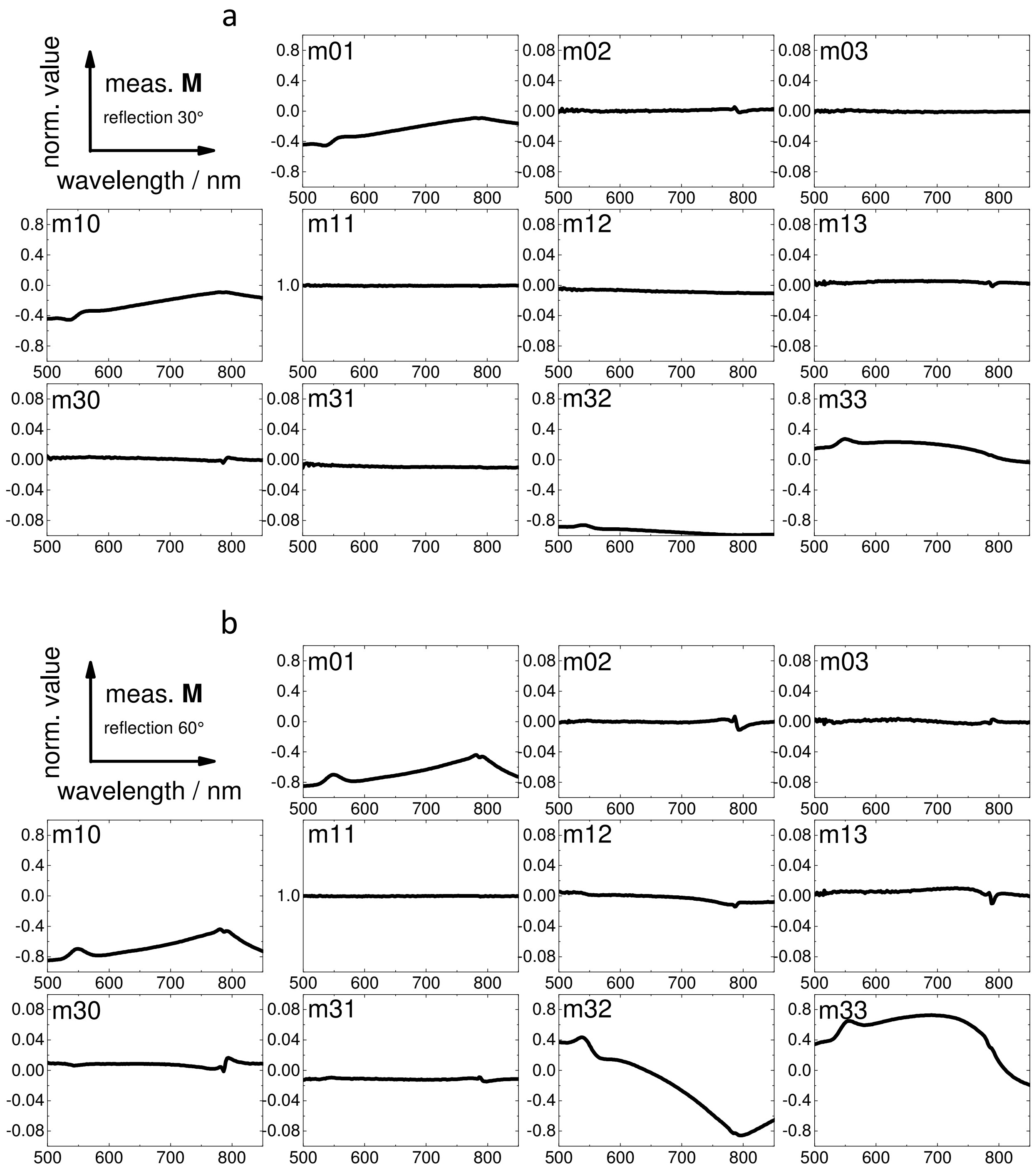

Supplementary Figure 8: As measured Mueller matrix data for reflection measurements on a 57 nm thick (*R,R*)-ProSQ-C16 thin film sample annealed at 180 °C under (a) 30° and (b) 60° angle of incidence.

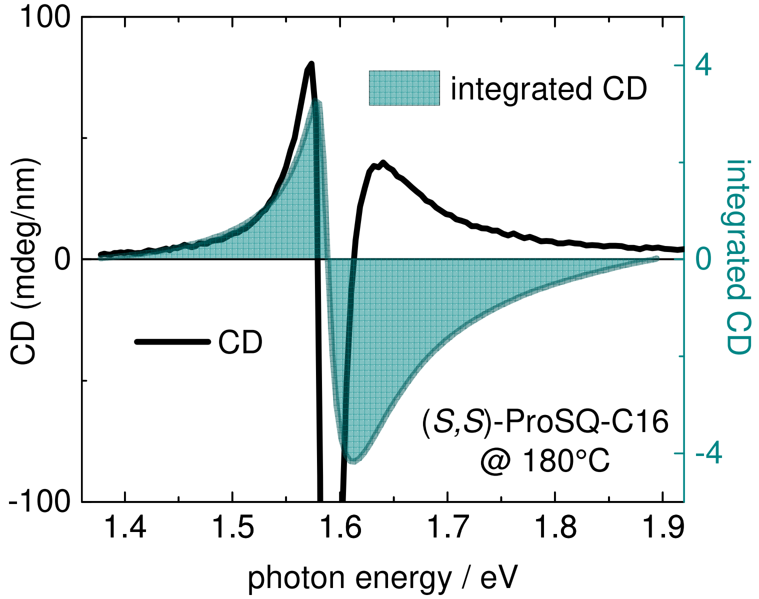

Supplementary Figure 9: Spectrally integrated circular dichroism: Circular dichroism versus photon energy (black line) of an (*S*,*S*)-ProSQ-C16 sample annealed at 180 °C and the spectral integral (cyan line/area) over the signal. The integration starts at 1.4 eV and the cyan curve displays the mathematical area underneath the CD curve (black line). The cyan shading is for illustration purposes. When the upper bound of integration reaches 1.9 eV the integral becomes zero. Thus, when the wider side peaks are included, the spectral integral approaches zero indicative for an excitonic CD response.

Supplementary Note 3: Reflection-Corrected Absorbance and Calculation of the Dissymmetry Factor

The model for the reflection corrected absorbance Abs^{cor} according to Eq. 7 (main paper) is simple and describes a free-standing film not including interference. Therefore we cross-check the validity of our approach by calculating the absorbance with more complex models for a free-standing film accounting for interference:

$$-\log(T) = -\log\left[\frac{(1-R)^2 \cdot e^{-\alpha d}}{1 - R^2 \cdot e^{-2\alpha d}}\right] \tag{1}$$

as well as for a film supported on a thick substrate accounting for interference, see Reference.[2] The optical constants provided in Fig. 3 (main paper) are used for the calculations. The absorbance versus layer thickness d at 780 nm for all three models is plotted together with the measured absorbance in Supplementary Fig. 10. Obviously, none of the models fully describes the measured data over the complete thickness range. However, all models provide the same slope of absorbance versus thickness when excluding very thin layer thicknesses. The simple approach is also valid for measurements from both sides of the sample, see Supplementary Fig. 7, since CD and absorbance are equal under normal incidence for both sample orientations. Thus there is no gain in applying more complex models based on wave-optics.

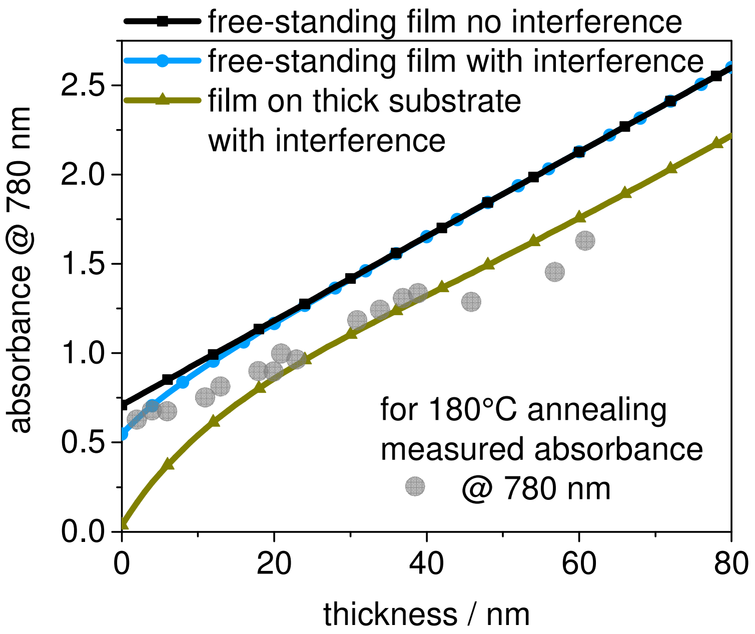

Supplementary Figure 10: Calculated absorbance versus layer thickness for (black square line) a free-standing film not accounting for interference, ((blue circle line) free-standing film including interference, Eq. (1), and (green triangle line) a film supported on a thick substrate accounting for interference, Ref.[2]. The grey circles are the measured absorbance of ProSQ-C16 samples annealed at 180 °C.

Supplementary Note 4: Thickness dependent T, R, A plus Calculation

In the Supplementary Figs. 11(a) and (b) measured transmission and reflection spectra are shown for ProSQ-C16 thin film samples with varying layer thicknesses. For layers thicker than 40 nm a characteristic dip within the reflection maximum becomes noticeable. The absorption is calculated from by $A = 1 - R - T$ and plotted in Supplementary Fig. 11(c). Obviously, around 780 nm the decrease in transmission is due to an increase in reflection rather than an increase in absorption. At 560 nm the decrease in transmission is caused rather by an increase in absorption as expected for an organic thin film. In Supplementary Figs. 11(d) and (e), the calculated transmission T, reflection R, and absorption A of a thin ProSQ-C16 film on glass as a function of its film thickness are presented at two characteristic spectral positions, 560 nm and 780 nm, respectively. For this the effective optical constants of ProSQ-C16 determined by spectroscopic ellipsometry, see Supplementary Fig. 5, and of the glass substrate are used. Transmission, reflection, and absorption of a thin organic film with multiple, coherent internal reflections on a thick substrate with incoherent internal reflections have been calculated.[2] In the thickness range from 0 nm to 100 nm, interference effects are mostly obvious at a wavelength of 780 nm due to the large value of the refractive index n. The calculations support the experimental findings presented in Supplementary Figs. 11(a) to (c).

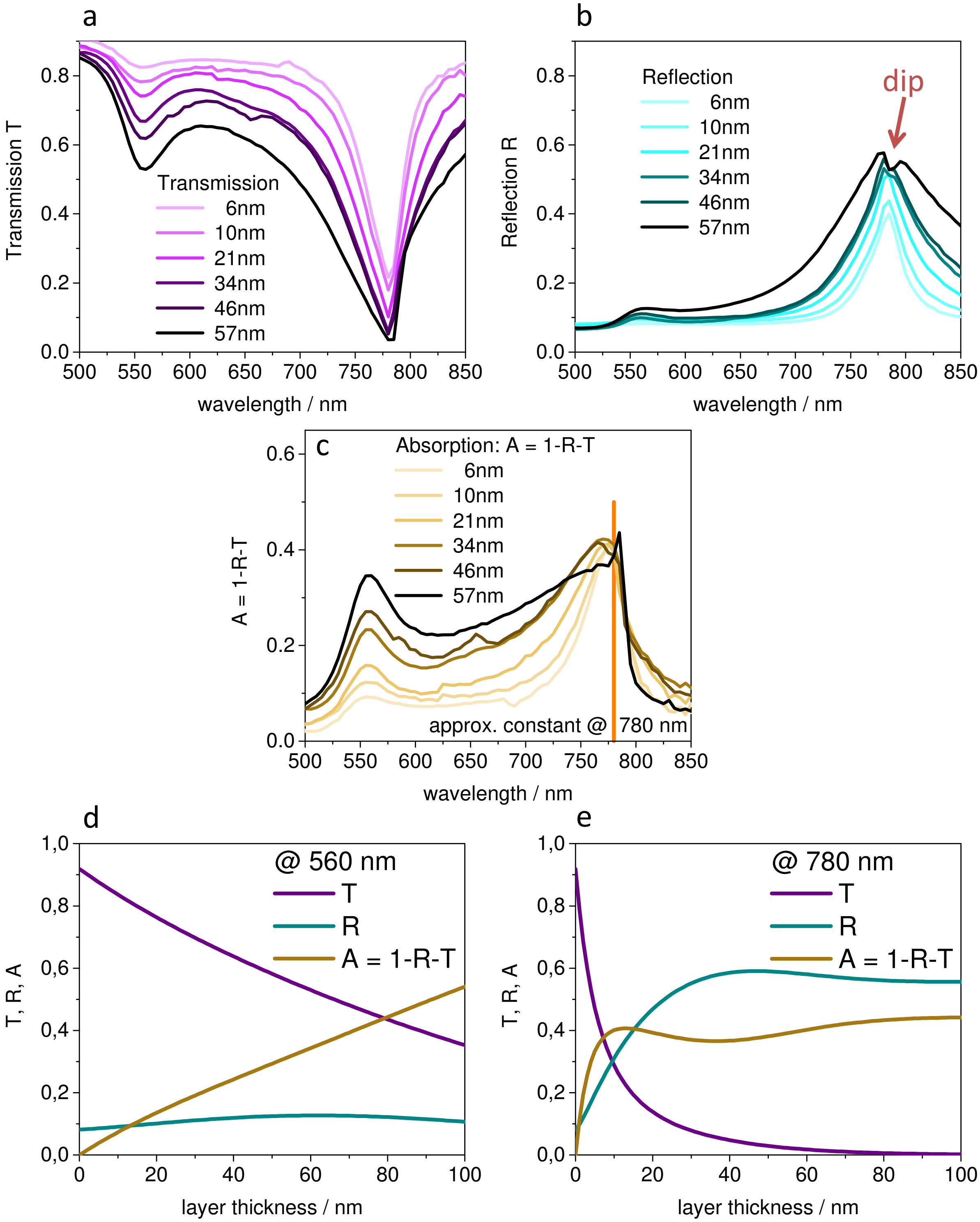

Supplementary Figure 11: Measured (a) transmission T under normal incidence and (b) specular reflection R under an angle of incidence of 15° except spectrum for 57 nm which is total reflection under 8° angle of incidence. For layers thicker than 40 nm a dip in the reflection spectrum is noticeable. (c) Calculated absorption: $A = 1 - T - R$. Calculated transmission, reflection, and absorption at (d) 560 nm and at (e) 780 nm.

Supplementary Note 5: Benchmarking of Circular Dichroism and Dissymmetry Factors for Organic Thin Film Samples and Metamaterials

Supplementary Table 1: Benchmarking

material	processing	λ (nm)	ellipticity (mdeg/nm)	g-value	method	year published
**(*R*,*R*)-<br>(*S*,*S*)-<br>ProSQ-<br>C16**	spincoating<br>+ thermal<br>annealing<br>180 °C	780	500<br>-325<br>note: incorrect linear approximate for purposes of comparison	0.75 ± 0.07<br>-0.45 ± 0.05	Mueller matrix spectroscopy	this work
**(*S*,*S*)-<br>ProSQ-<br>C16<br>(*S*,*S*)-<br>ProSQ-C6**	spincoating<br>+ thermal<br>annealing<br>120 °C	780<br>(540)	-15<br>(*NA*)<br>-9<br>(-19)	-0.026<br>(*NA*)<br>-0.039<br>(-0.032)	Jasco J-810<br>range limitation, not accounting for artifacts	previous work 2017[1]
**(*S*,*S*)-<br>NPTTPN**	spincoating<br>+ thermal<br>annealing<br>60 °C	440	-1	$-5 \cdot 10^{-3}$	Mueller matrix spectroscopy	this work, Supplementary information
polythiophene	spincoating	600	*NA*	$4 \cdot 10^{-3}$	Jasco J-600	2007[3]
polythiophene<br>**PBMBT**	spincoating	600	*NA*	$7 \cdot 10^{-3}$<br>$2 \cdot 10^{-2}$ at low temperature	Jasco J-600	2000[4]
poly-(phenylene)-bithiophene	spincoating<br>+ thermal<br>annealing	600	*NA*	$7 \cdot 10^{-3}$<br>$2 \cdot 10^{-2}$ after annealing, described as pseude CD	Jasco J-810, samples rotated	2007[5]
oligothiophene	dropcasting	299<br>351<br>420	*NA*	$\pm 7.6 \cdot 10^{-3}$<br>$\pm 1.3 \cdot 10^{-2}$<br>$\pm 1.8 \cdot 10^{-3}$<br>inversion upon sample flipping	Jasco J-710<br>samples flipped	2017[6]

Supplementary Table 1: Benchmarking

material	processing	λ (nm)	ellipticity (mdeg/nm)	g-value	method	year published
polyfluorene **c-PFBT**	spincoating + thermal annealing 240 °C	500	true: 2 for a 20 nm film estimated from [7]	thickness dependent up to -0.8, effect is due to cholesteric ordering, not intrinsic CD; true g-value 20 nm film: 0.001 from previous paper [7]	Mueller matrix spectroscopy	2017 [8]
fluorene-based copolymers<br>**PF**<br>**PFPh**<br>**PFTh**<br>**PFBT**	spincoating + thermal annealing 120 °C	409<br>393<br>436, 480<br>485, 351	*NA*<br>30<br>6<br>32<br>estimated from Fig.s 9 and 6 44 nm, 80 nm, 60 nm thick films	 -0.25<br>0.15<br>0.02, -0.03<br>0.36, 0.18<br>calculated from Tab. 1	Jasco J-815	2016 [9]
polyfluorenes<br>**LaPPS61**<br>**LaPPS62**	spincoating + thermal annealing 200 °C	400<br>455	2<br>estimated from Fig. 2<br>12<br>estimated from Fig. 4	0.018<br>0.09<br>from Fig. 5, thickness dependent g	Jasco J-715	2013 [10]
polyfluorenes<br>**PFP1**<br>**PFP2**<br>**PFP3**	spincoating (+ thermal annealing)	380	-1.4<br>-1.9 / (-13)<br>-6.3<br>estimated from Fig. 2(a) and layer thicknesses 37, 53, 67 nm	*NA*<br>-0.002 / (-0.2)<br>-0.055 / (-0.8)<br>note: mesoscopic ordering effects are present	Jasco J-815 measurement range exceeded	2016 [11]
neomenthyl-polyfluorenes **Alt.**	spincoating + thermal annealing 160 °C	393	0.5 – 2 (600 mdeg from Fig. 3 thickness 330 nm – 1310 nm from SI)	0.026<br>note: significant LD Fig. S2(E)	Jasco J-820, sample rotation	2014 [12]
helicene-like molecule **8**	pulsed laser deposition	230	±2.2 estimated from Fig. 15 for a 90 nm thick film	$\pm 4 \cdot 10^{-3}$ estimated from Fig. 1	homemade spectrometer	2016 [13]

Supplementary Table 1: Benchmarking

material	processing	λ (nm)	ellipticity (mdeg/nm)	g-value	method	year published
tetra-substituted paracyclophanes **R-CP3** **S-CP3** **R-CP5** **S-CP5**	spincoating or dropcasting + thermal annealing 65 °C or 90 °C	**CP3**: 350 **CP5**: 400	*NA*	spin/drop: -0.088/-0.017 +0.013/+0.016 -0.15/-0.15 +0.11/+0.15 note: g for dropcasted samples estimated by authors	Jasco J-820	2017 [14]
perylene diimides **S/R-CPDI-Ph**	solution-crystallized nanowires, thermally evaporated thin films	450	*NA* ±0.15 estimated from Fig. S8	$\pm1.5 \cdot 10^{-3}$ $\pm4.0 \cdot 10^{-4}$ estimated from Fig. 3	Jasco J-815	2017 [15]
poly-para-phenylene-ethynylene	spincoating + thermal annealing 160 °C	432	130-240 estimated from Fig. 1 and layer thicknesses 90 nm to 50 nm	-0.38 related to mesoscopic structure formation	OLIS RSM 1000 CD spectrophotometer, samples rotated and flipped	2002 [16]
liquid crystalline polyacetylenes **R-PA2**	spincoating + chiral dopant	(378) 455	*NA*	($1.5 \cdot 10^{-4}$ solution) $3.7 \cdot 10^{-2}$ interchain pi-stacking $1.7 \cdot 10^{-1}$ lyotropic film with helical order due to chiral dopant	Jasco J-820	2012 [17]
poly-para-phenylenes **R/S-PPP1-SNap**	spincoating	380	*NA*	10^{-2} – 10^{-1}	*NA*	2015 [18]

Supplementary Table 1: Benchmarking

material	processing	λ (nm)	ellipticity (mdeg/nm)	g-value	method	year published
twisted bilayer graphene	complex preparation	335	6.5	*NA*	alternating left- and right-CP light illumination	2016 [19]
plasmonic nanohelix	dynamic physical deposition growth	711	60 metamaterial pseudo-CD	*NA*	*NA*	2013 [20]
planar metal structure	e-beam lithography	790	10 metamaterial pseudo-CD	*NA*	*NA*	2005 [21]

Chirally substituted polyfluorenes and also poly-para-phenylene-ethynylenes appear superior but the strong circular dichroism is due to mesoscopic ordering, i.e., cholesteric liquid crystalline ordering and, therefore is not *true* CD based on magneto-electric origin. Poly- and oligo-thiophenes do not show mesoscopic structural ordering, thus their g-values are on the order of 10^{-3} – 10^{-2} as typical for organic semiconductors. Small molecular or oligomeric based examples are the perylene bisimides and the tetra-substituted paracyclophanes, respectively. The former have g-values three orders of magnitude below and the latter a factor of 5 below our annealed ProSQ-C16 thin films. However, our previous experiments conducted with a conventional CD spectropolarimeter clearly underestimate the circular dichroic properties of the material illustrating how critical the *quantitative* measurement of *true* circular dichroism is.

Supplementary Note 6: Benchmarking: (*S,S*)-NPTTPN

The phenyl-bithiophene with chiral dimethylethylamine functional groups (1*S*,1'*S*)-1,1'-([2,2'-bithiophene]-5,5'-diylbis(4,1-phenylene))bis(*N,N'*-dimethylethylamine) shortly named (*S,S*)-NPTTPN has been synthesized by us as outlined below. It is used as reference material for circular dichroism in thin films samples, which were prepared and measured under the same

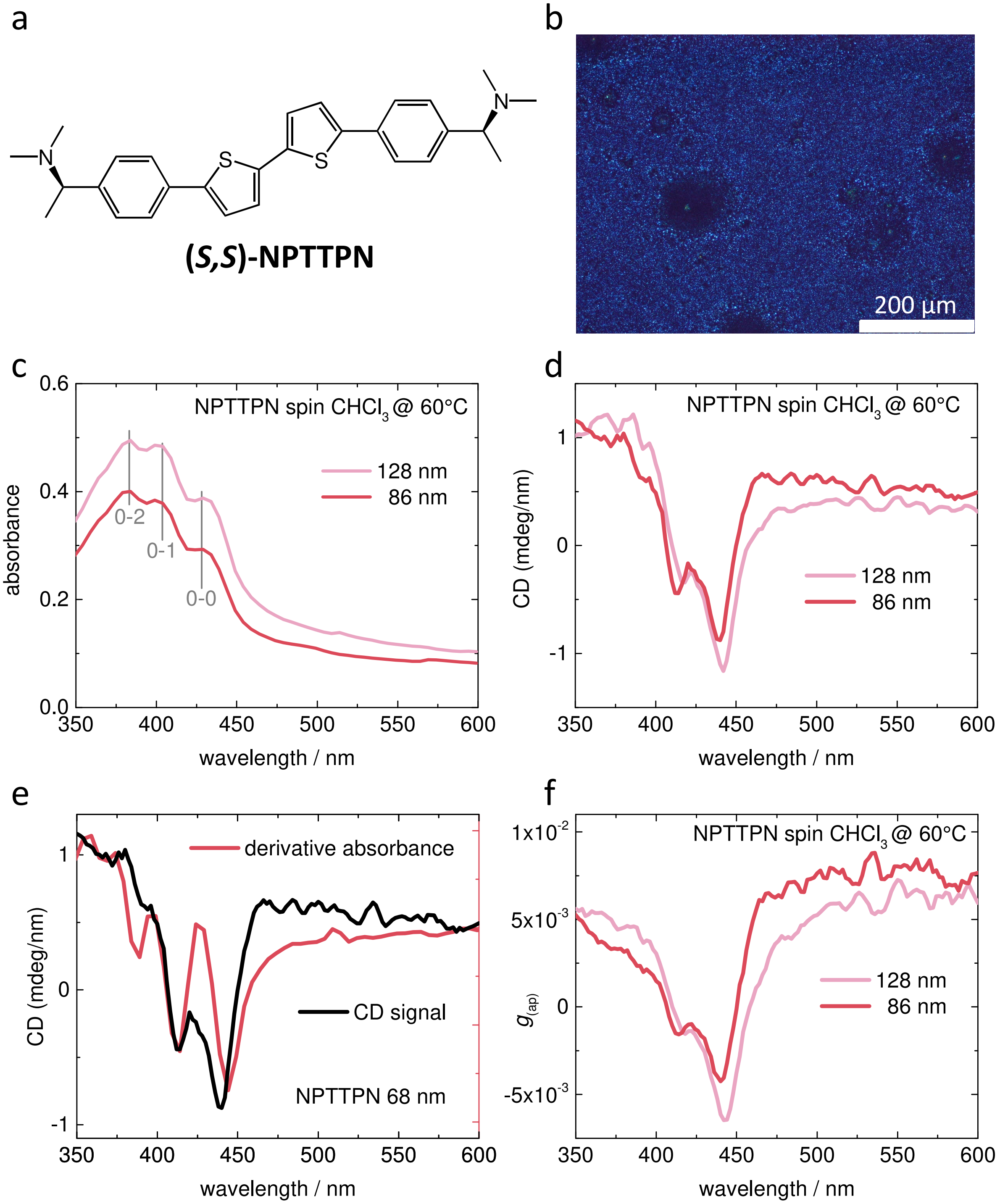

Supplementary Figure 12: (a) Structural formula of (*S,S*)-NPTTPN. Characterization of (*S,S*)-NPTTPN thin films annealed at 60 °C with varying layer thicknesses (86 nm and 128 nm): (b) Overexposed microscopy image between crossed polarizers. (c) Absorbance spectra. (d) Thickness normalized circular dichroism spectra. (e) Thickness normalized CD (black curve) and derivative of absorbance spectrum (red curve). (f) Apparent dissymmetry factor g_{ap}.

conditions as the ProSQ-C16 samples, see Methods in the main paper. The structural formula of (*S,S*)-NPTTPN is shown in Supplementary Fig. 12(a). It was chosen because it is similar to the ProSQ-C16 in the sense that it is a rod-like molecular semiconductor with an amine-based terminal homochiral functional group being on both ends of the molecule. Upon spincoating of (*S,S*)-NPTTPN from a chloroform solution and subsequent thermal annealing a featureless film is formed with faint birefringence. Thus, no amplification of the CD is expected due to mesoscopic structural ordering, just as in case of the ProSQ-C16. An overexposed microscopy image between crossed polarizers after annealing at 60 °C is displayed in Supplementary Fig. 12(b). Note that higher annealing temperatures did not induce crystallization, and neither enhanced the circular dichroism, therefore we show the best data obtained for the 60 °C annealing.

The absorbance spectra in Supplementary Fig. 12(c) of NPTTPN films with varying layer thicknesses show typical spectral signatures of weakly coupled molecular H-aggregates with pronounced vibronic transitions.[22] These vibronic progressions are also noticeable in the circular dichroism spectra displayed in Supplementary Fig. 12(d). The CD spectrum is a superposition of bisignate bands for each vibronic progression, which has also been documented for polythiophene thin films.[4] The CD spectrum has a bisignated appearance with a positive lobe at shorter wavelength and a negative lobe at longer wavelength and the maximum CD values approximate to ± 1 mdeg/nm. The CD spectrum is proportional to the derivative of the absorbance spectrum, see Supplementary Fig. 12(e), clearly indicating an excitonic CD signal. Finally, in Supplementary Fig. 12(f) the absorbance normalized apparent dissymmetry factor g_{ap} is plotted revealing maximum values of $\pm 5 \cdot 10^{-3}$ which is a typical value for polythiophene[3,4], poly(phenylene)bithiophene[5] and oligothiophene[6] thin films, see also Supplementary Tab. 1 above. Thus, the excitonic origin of the CD signal alone is not sufficient to boost the g-value.

We refrain from calculating the true g-value, g_{true}, since we would need additional samples to perform the reflection-correction. More substantially, due to the vibronic progressions, we

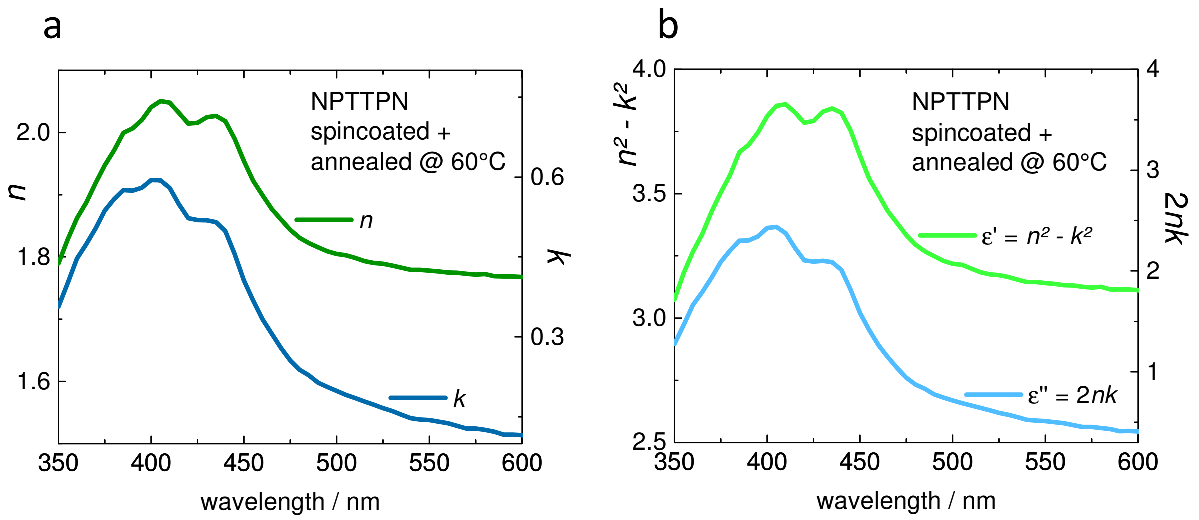

Supplementary Figure 13: (a) Effective optical constants and (b) real and imaginary part of the dielectric function of a NPTTPN thin film.

would need to refine the concept of our proposed reflection-correction, which goes beyond the scope of the benchmarking. Note that our molecule of main interest, the ProSQ-C16, does not show vibronic progressions due to strong intermolecular interactions. This is the marked difference of NPTTPN and ProSQ-C16: while NPTTPN forms weakly coupling molecular aggregates the ProSQ-C16 forms strongly coupling molecular aggregates. However, we clearly demonstrate by the analysis of (S,S)-NPTTPN that typical excitonic CD signals (for weak excitonic coupling) are by a factor of 150 smaller than the maximum CD values of our new benchmark material ProSQ-C16.

Effective optical constants have been determined by spectroscopic ellipsometry as described in the Methods section of the main paper. Here in addition to the described procedure, surface roughness needed to be taken into account by an effective medium layer with 50 % voids. In Supplementary Fig. 13 (a) the optical constants and in Supplementary Fig. 13 (b) the real and imaginary part of the dielectric function of a NPTTPN thin film annealed at 60 °C are displayed. The values of the optical constants are typical for an organic semiconductor but rather on the lower edge.[23] The oscillator strength is certainly not large enough to translate into a negative real part of the dielectric function as it is the case for the strongly coupling ProSQ-C16 material. Thus, no "excitonic amplification" of the circular dichroism

for the weak excitonic coupling of the NPTTPN molecular aggregates can be expected.

Synthesis and Analytical Details of (*S,S*)-NPTTPN

A 50 mL two-necked flask equipped with a condenser was charged with 272 mg (0.66 mmol) 5,5'-diiodo-2,2'-bithiophene, 400 mg (1.45 mmol, 2.2 equivalents), (*S*)-1-(4-*N,N'*-dimethyl-ethylamine)phenylboronic acid pinacol ester, 840 mg (3.96 mmol, 6 equivalents) potassium phosphate, 60 mg (0.066 mmol, 10 mol%) tris(dibenzylideneacetone)dipalladium(0) ($Pd_2(dba)_3$), and 91 mg (0.165 mmol, 25 mol%) 1,1'-bis(diphenylphosphino)ferrocene (dppf) under an argon atmosphere. After adding 14 mL 1,4-dioxane and 3.5 mL water the reaction mixture was degassed and heated to 100 °C for 42 h under an argon atmosphere. The water phase was extracted three times with dichloromethane, and the combined organic phase was dried over sodium sulphate. The solvent was removed under reduced pressure and the remaining crude product was purified by column chromatography on silica gel using cyclohexane:ethyl acetate (1:5) plus 5 % triethyl amine as an eluent, R_f factor: 0.25.

Yield: 125 mg (60 %)

Molecular formula: $C_{28}H_{32}N_2S_2$

Molar mass: 460.70 g/mol

Optical rotation: $[\alpha]_D^{20} = -99.0°$ in dichloromethane 0.5 mg/mL

UV/Vis: $[\lambda]_{max}^{ex} = 378$ nm in dichloromethane

Fluorescence: $[\lambda]_{max}^{em} = 460$ nm in dichloromethane

ESI^+ HRMS (Bruker microTOF-Q): m/z calcd for $C_{28}H_{32}N_2S_2H$ $[M+H]^+$: 461.2080, found: 461.2080.

Elemental analysis (Heraeus Vario EL): $C_{28}H_{32}N_2S_2 \cdot CH_2Cl_2$ calcd C: 71.93, H: 6.92, N: 5.97, S: 13.67; found: C: 72.12, H: 6.58, N: 5.88, S: 13.46.

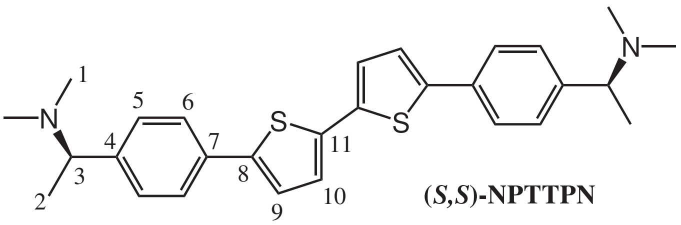

(*S,S*)-NPTTPN

^{1}H NMR (Bruker DPX 400): (400 MHz, CD_2Cl_2, RT) δ [ppm] = 7.57 (d, J = 8.2 Hz, 4 H, H-6), 7.34 (d, J = 8.2 Hz, 4 H, H-5), 7.26 (d, J = 3.8 Hz, 2 H, H-9), 7.19 (d, J = 3.8 Hz, 2 H, H-10), 3.26 (q, J = 6.7 Hz, 2 H, H-3), 2.18 (2, 12 H, H-1), 1.34 (d, J = 6.7 Hz, 6 H, H-2).

^{13}C NMR (Bruker DPX 400): (100 MHz, CD_2Cl_2, RT) δ [ppm] = 145.1 (C-4), 143.6 (C-7), 136.9 (C-11), 133.0 (C-8), 128.7 (C-5), 125.9 (C-6), 125.0 (C-10), 124.1 (C-9), 66.0 (C-3), 43.5 (C-1), 20.4 (C-2).

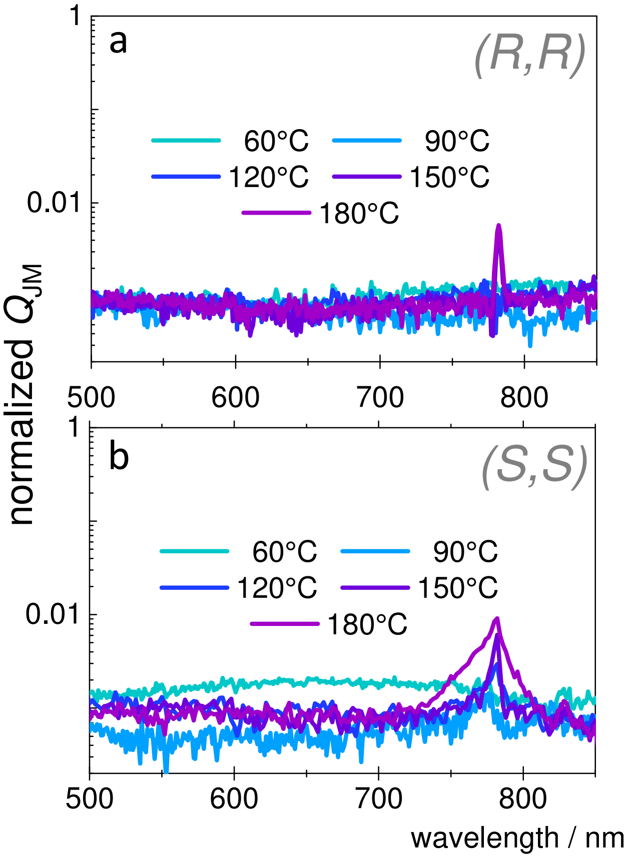

Supplementary Figure 14: The Jones matrix quality factor Q_{JM} ranges from 0 to a maximum of $1/\sqrt{5} \approx 0.447$ for a fully depolarizing sample.[24] Here it is normalized to be 1 for full depolarization for Mueller matrix scans of (a) (R,R)-ProSQ-C16 and (b) (S,S)-ProSQ-C16 thin films annealed at the indicated temperatures. Small depolarization effects occur where the extinction has its maximum, but remain well below 1 %, and thus are considered insignificant.

Supplementary References

1. Schulz, M.; Mack, M.; Kolloge, O.; Lützen, A.; Schiek, M. Organic Photodiodes from Homochiral L-Proline Derived Squaraine Compounds with Strong Circular Dichroism. *Phys. Chem. Chem. Phys.* **2017**, *19*, 6996–7008.

2. Stenzel, O. In *The Physics of Thin Film Optical Spectra*, 2nd ed.; Ertl, G., Lüth, H., Mills, D., Eds.; Springer Series in Surface Sciences; Springer: Berlin, 2016; Vol. 44.

3. Lakhwani, G.; Koeckelberghs, G.; Meskers, S. C. J.; Janssen, R. The chiroptical properties of chiral substituted poly[3-((3S)-3,7-dimethyloctyl)thiophene] as a function of film thickness. *Chem. Phys. Lett.* **2007**, *437*, 193–197.

4. Langeveld-Voss, B.; Janssen, R.; Meijer, E. On the origin of optical activity in polythiophenes. *J. Mol. Struct.* **2000**, *521*, 285–301.

5. Koeckelberghs, G.; Vangheluwe, M.; Persoons, A.; Verbiest, T. Chirality in Poly(phenylene-*alt*-bithiophene)s: A Comprehensive Study of Their Behavior in Film and Nonsolvents. *Macromolecules* **2007**, *40*, 8142–8150.

6. Albano, G.; Lissia, M.; Pescitelli, G.; Aronica, L. A.; Bari, L. D. Chiroptical response inversion upon sample flipping in thin films of a chiral benzo[1,2-*b*:4,5-*b'*]dithiophene-based oligothiophene. *Mater. Chem. Front.* **2017**, *1*, 2047–2056.

7. Lakhwani, G.; Meskers, S. C. J. Insights from chiral polyfluorene on the unification of molecular exciton and cholesteric liquid crystal theories for chiroptical phenomena. *J. Phys. Chem. A* **2011**, *116*, 1121–1128.

8. Di Nuzzo, D.; Kulkarni, C.; Zhao, B.; Smolinsky, E.; Tassinari, F.; Meskers, S. C.; Naaman, R.; Meijer, E.; Friend, R. H. High circular polarization of electroluminescence achieved via self-assembly of a light-emitting chiral conjugated Polymer into multidomain cholesteric films. *ACS Nano* **2017**, *11*, 12713–12722.

9. Cho, M. J.; Ahn, J.-S.; Kim, Y.-U.; Um, H. A.; Prasad, P. N.; Lee, G. J.; Choi, D. H. New fluorene-based chiral copolymers with unusually high optical activity in pristine and annealed thin films. *RSC Adv.* **2016**, *6*, 23879–23886.

10. Nowacki, B.; Oh, H.; Zanlorenzi, C.; Jee, H.; Baev, A.; Prasad, P. N.; Akcelrud, L. Design and synthesis of polymers for chiral photonics. *Macromolecules* **2013**, *46*, 7158–7165.

11. Lee, G. J.; Choi, E. H.; Ham, W. K.; Hwangbo, C. K.; Cho, M. J.; Choi, D. H. Circular dichroism, surface-enhanced Raman scattering, and spectroscopic ellipsometry studies of chiral polyfluorene-phenylene films. *Opt. Mater. Express* **2016**, *6*, 767–781.

12. Watanabe, K.; Koyama, Y.; Suzuki, N.; Fujiki, M.; Nakano, T. Gigantic chiroptical enhancements in polyfluorene copolymers bearing bulky neomenthyl groups: importance of alternating sequences of chiral and achiral fluorene units. *Polym. Chem.* **2014**, *5*, 712–717.

13. Bensalah-Ledoux, A.; Pitrat, D.; Reynaldo, T.; Srebro-Hooper, M.; Moore, B.; Autschbach, J.; Crassous, J.; Guy, S.; Guy, L. Large-Scale Synthesis of Helicene-Like Molecules for the Design of Enantiopure Thin Films with Strong Chiroptical Activity. *Chem. Eur. J.* **2016**, *22*, 3333–3346.

14. Gon, M.; Sawada, R.; Morisaki, Y.; Chujo, Y. Enhancement and controlling the signal of circularly polarized luminescence based on a planar chiral tetrasubstituted [2.2]Paracyclophane framework in aggregation system. *Macromolecules* **2017**, *50*, 1790–1802.

15. Shang, X.; Song, I.; Ohtsu, H.; Lee, Y. H.; Zhao, T.; Kojima, T.; Jung, J. H.; Kawano, M.; Oh, J. H. Supramolecular Nanostructures of Chiral Perylene Diimides with Amplified Chirality for High-Performance Chiroptical Sensing. *Adv. Mater.* **2017**, *29*, 1605828.

16. Wilson, J. N.; Steffen, W.; McKenzie, T. G.; Lieser, G.; Oda, M.; Neher, D.; Bunz, U.

H. F. Chiroptical Properties of Poly(*p*-phenyleneethynylene) Copolymers in Thin Films: large *g*-Values. *J. Am. Chem. Soc.* **2002**, *124*, 6830–6831.

17. San Jose, B. A.; Matsushita, S.; Akagi, K. Lyotropic chiral nematic liquid crystalline aliphatic conjugated polymers based on disubstituted polyacetylene derivatives that exhibit high dissymmetry factors in circularly polarized luminescence. *J. Am. Chem. Soc.* **2012**, *134*, 19795–19807.

18. Watanabe, K.; Sun, Z.; Akagi, K. Interchain Helically π-Stacked Assembly of Cationic Chiral Poly (para-phenylene) Derivatives Enforced by Anionic π-Conjugated Molecules through Both Electrostatic and π–π Interactions. *Chem. Mater.* **2015**, *27*, 2895–2902.

19. Kim, C.-J.; Sánchez-Castillo, A.; Ziegler, Z.; Ogawa, Y.; Noguez, C.; Park, J. Chiral atomically thin films. *Nat. Nanotechnol.* **2016**, *11*, 520–524.

20. Gibbs, J.; Mark, A.; Eslami, S.; Fischer, P. Plasmonic nanohelix metamaterials with tailorable giant circular dichroism. *Appl. Phys. Lett.* **2013**, *103*, 213101.

21. Kuwata-Gonokami, M.; Saito, N.; Ino, Y.; Kauranen, M.; Jefimovs, K.; Vallius, T.; Turunen, J.; Svirko, Y. Giant optical activity in quasi-two-dimensional planar nanostructures. *Phys. Rev. Lett.* **2005**, *95*, 227401.

22. Spano, F. C. The Spectral Signatures of Frenkel Polarons in H- and J-Aggregates. *Acc. Chem. Res.* **2010**, *43*, 429–439.

23. Vezie, M. S.; Few, S.; Meager, I.; Pieridou, G.; Dörling, B.; Ashraf, R. S.; Goñi, A. R.; Bronstein, H.; McCulloch, I.; Hayes, S. C. *et al.* Exploring the origin of high optical absorption in conjugated polymers. *Nat. Mater.* **2016**, *15*, 746–753.

24. Hilfiker, J. N.; Hale, J. S.; Herzinger, C. M.; Tiwald, T.; Hong, N.; Schöche, S.; Arwin, H. Estimating depolarization with the Jones matrix quality factor. *Appl. Surf. Sci.* **2016**, *421, Part B*, 494–499.
