# Supplementary material for: Giant intrinsic circular dichroism of prolinol-derived squaraine thin films
Source: Nat Commun. 2018 Jun 20;9:2413. doi: 10.1038/s41467-018-04811-7 (PMC6010436; doi:10.1038/s41467-018-04811-7)
Supplement: Supplementary file 3 — Description of Additional Supplementary Files [file 41467_2018_4811_MOESM3_ESM.pdf]

Description of Additional Supplementary Files

File Name: Supplementary Movie 1
Description: Bireflectance of a spin-casted (R,R)-ProSQ-C16 film on glass, annealed at 180°C. The series of images was taken in reflection for white light illumination with a Leica DMRME optical microscope, where the sample was rotated in steps of 5°. The cross indicates the direction of polarizer (white) and analyzer (orange).

File Name: Supplementary Movie 2
Description: Bireflectance of a spin-casted (S,S)-ProSQ-C16 film on glass, annealed at 180°C. The series of images was taken in reflection for white light illumination with a Leica DMRME optical microscope, where the sample was rotated in steps of 5°. The cross indicates the direction of polarizer (white) and analyzer (orange).
